# Reasons to decrease or stop nicotine and tobacco use among adults and association with MPOWER scores in twenty-one middle- and high-income countries, 2019–2020

**DOI:** 10.18332/tpc/167957

**Published:** 2023-07-21

**Authors:** Saida R. Sharapova, Carrie Whitney, Rose Sulentic, Liping Pan, Indu B. Ahluwalia

**Affiliations:** 1 Independent Researcher, Atlanta, United States; 2 CDC Foundation, Atlanta, United States; 3 Global Tobacco Control Branch, Office on Smoking and Health, National Center for Chronic Disease Prevention and Health Promotion, Centers for Disease Control and Prevention, Atlanta, United States

**Keywords:** cessation, MPOWER, tobacco, reasons to quit

## Abstract

**INTRODUCTION:**

This study examined reasons why people planned to reduce or stop tobacco consumption and their relationship with MPOWER scores, adjusting for sociodemographic, cultural, and economic factors.

**METHODS:**

Data used were Euromonitor International’s Voice of the Consumer: Nicotine Survey 2019–2020, World Bank’s country income and WHO’s MPOWER policy scores. Analytical sample included 21913 adults of legal smoking age in 21 middle- and high-income countries who used nicotine and tobacco products and planned to reduce or stop their consumption in the next 12 months. Poisson regression models with robust error variance, adjusted for sociodemographic and tobacco use covariables, generated adjusted risk ratios (ARRs) of selecting a certain reason to reduce tobacco consumption dependent on continuous MPOWER scores.

**RESULTS:**

Main reasons to reduce or stop tobacco consumption were improving health (85%), saving money (65%), pressure from family (19%), and using another substance instead (4%). Country variation was observed by MPOWER scores. Positive associations were found between some MPOWER scores and reasons to reduce or stop tobacco consumption: enforcing bans on tobacco advertising and using another substance (ARR=1.28; 95% CI: 1.11–1.47); warning about dangers of tobacco and saving money (ARR=1.25; 95% CI: 1.19–1.32); offering help to quit tobacco and using another substance (ARR=1.26; 95% CI: 1.10–1.45) or family pressure (ARR=1.11; 95% CI: 1.04–1.17); anti-tobacco campaigns and using another substance (ARR=1.15; 95% CI: 1.08–1.23); and raising taxes and saving money (ARR=1.11; 95% CI: 1.09–1.13).

**CONCLUSIONS:**

MPOWER scores are associated with reported reasons to quit tobacco including to improve health, save money, respond to family pressure or use another substance instead.

## INTRODUCTION

Globally, tobacco use is the leading cause of preventable disease and death, killing more than 8 million people yearly^1^. The annual economic burden of tobacco use is US$1.4 trillion^2^. The majority of people who use tobacco smoke cigarettes^3^. However, global market sales of nicotine products, including e-cigarettes and heated tobacco products, are growing^4^. The majority of people who use cigarettes, e-cigarettes or smokeless tobacco want to quit^5,6^. Understanding why people want to quit is important to tailor services and policies supporting cessation^7,8^. In 2008, the World Health Organization (WHO) introduced the MPOWER policy package to help policy makers at the country level implement six effective measures to reduce the demand for nicotine and tobacco products (NTPs) and promote cessation of NTPs consumption: Monitor tobacco use; Protect people from tobacco smoke; Offer to help quit tobacco use; Warn about the dangers of tobacco, including anti-tobacco mass media campaigns; Enforce tobacco advertising, promotion, and sponsorship bans; and Raise taxes on tobacco^9^. Motivation and effective programs to change behavior are critical to tobacco cessation^10^. Previous studies have examined motivations to quit tobacco and found the most commonly cited reasons are related to health concerns, costs, and social pressure^11-13^. The MPOWER measures of protect, offer, warn, raise, including campaigns, may target individual motivation to quit tobacco more directly than the other measures^2^. This study examines reasons why people who use NTPs would like to reduce or completely stop their consumption in the next 12 months and if these reasons differ by the scale of MPOWER policies when adjusted for social, cultural, economic, and demographic factors. The data are standardized, international, and reflect recent NTPs market information, allowing for country-to-country comparisons over a two-year period.

## METHODS

This ecologic study was based on secondary data analysis of data collected by the Voice of the Consumer: Nicotine Survey (Nicotine Survey) data. This is an annual online opt-in survey of adult participants on panels in selected countries conducted by Euromonitor International, Ltd. The survey was conducted in 2019 and 2020 in Canada, China, Czech Republic, France, Germany, Greece, Israel, Italy, Japan, Kazakhstan, Netherlands, Poland, Romania, the Russian Federation (Russia), Slovakia, South Korea, Spain, Ukraine, the United Kingdom (UK), and the United States of America (USA). In 2020 only, the survey was also conducted in Australia. The survey was exempt from ethical review as it was an anonymous survey of adults who opted to participate in the survey, carried minimal risks, and made provisions to remove any personally identifying information from the data. The sample in each country was pre-screened to match the country’s population according to nested quotas for gender and age (adults of legal age to use tobacco: from 19 years in South Korea, 20 years in Japan, 21 years in USA, and 18 years for all remaining countries). Data included only unique, complete responses with response time appropriate for reading the questions and response options and unique and legible open-ended responses. In countries with <70% internet coverage rates, samples included 1750 online and 250 phone-based respondents (China, Italy, Romania, and Ukraine). There are 82864 respondents in the combined data (2019: n=40417; 2020: n=42447). The sample sizes varied from 2071 in Australia (2020 data only) to 4123 in China (2019–2020 data). The median sample size was 4030 respondents in the combined 2019–2020 data^14^.

Dependent variables were reasons reported by Nicotine Survey respondents who used NTPs at least monthly (n=32488) for why they plan to decrease or stop tobacco consumption in the next 12 months. Reasons provided by the survey as response options included improving health, saving money, pressure from the family, using another substance instead of NTPs, other, and none of the above. Respondents could select as many reasons as applied, so the selected reasons were not mutually exclusive (total number of responses was 23569). Due to the small sample size, response options ‘other’ (n=561) and ‘none of the above’ (n=295) yielded estimates with the relative standard error exceeding 30% and were omitted from analysis.

Independent variables included individual-level data from the Nicotine Survey and country-level variables data from the World Health Organization (WHO) and World Bank databases specified below.

### Nicotine survey variables

Nicotine and tobacco products included cigarettes, hand-rolled/roll-your-own/make-your-own tobacco (RYO), smokeless tobacco (SLT), cigars, cigarillos, shisha/hookah/pipe tobacco (shisha), e-cigarettes, and heated tobacco products (HTPs). Respondents indicated all NTPs they used and frequency of use for each: daily (at least once per day for the last month), weekly (at least once per week for the last month), monthly (at least once per month, for the last 6 months), yearly (at least once in the past 12 months), more than a year ago, or never used. Supplementary file Attachment 1 provides definitions of the NTPs.

NTP use was categorized as multiple product use if a respondent reported using more than one NTP at least monthly or categorized as use of a single NTP only if using any one NTP at least monthly. Single NTP only use was further separated into use of cigarettes, cigarillos, cigars, e-cigarettes, HTP, RYO, shisha, and SLT. Yearly use (n=1477) and using NTPs more than a year ago (n=300) were included with the ‘no or infrequent use’ category.

Other individual-level independent variables included sex (women, men), age group (18–24, 25–34, 35–44, 45–54, 55–64 and 65–74 years), nativity status (have lived in the country for <5 years, have lived in the country for ≥5 years, native born and undisclosed), living with children aged <18 year (yes or no), living with another person who uses NTPs (yes or no), living in rural or urban area, annual family income, education level, and social norms.

Annual income in the Nicotine Survey was collected in local currency units and converted into US$ by using an average conversion rate during the year of the survey. To understand the annual family income within a country’s context, we converted income ranges presented in US$ into International Dollars using the World Bank Purchasing Power Parities conversion factors for private consumption (2020) and categorized the income as below or at or above the national average annual household income for each respective country^15^. Education level varied by country and the highest level achieved was assessed as follows: primary school graduate, secondary school graduate, A-level or college equivalent (UK and France only), vocational school graduate or technical school certificate, associate’s or ‘2-year’ degree from junior college or technical school (USA only), Bachelor’s degree from college or university, graduate or postgraduate studies (e.g. Master’s, doctoral degree, and other).

A variable to assess social norms was created as a combination of positive or negative responses to the following statements: ‘It is considered normal among my friends and family to consume nicotine and tobacco products’; ‘Very few of my peer group consume tobacco or nicotine products’; and ‘My peers often consume nicotine and tobacco products when we are together’. If all three responses indicated supportive attitudes towards NTPs use, the variable was categorized as ‘tobacco use is normal’. If all three responses indicated support toward not using NTPs, the variable was categorized as ‘tobacco use is not normal’. One or two supportive responses were categorized as ‘tobacco use is somewhat normal’.

### Country-level variables

Countries were classified as middle-income (combined lower and upper middle) or high-income based on their gross national income per capita according to the World Bank data^16^. Summary (sum of all scores for a country) MPOWER scores were calculated for each survey country using the WHO MPOWER scores^17^ (Supplementary file Attachment 2).

### Statistical analysis

Analyses were conducted using Microsoft Excel (Microsoft® Excel® for Microsoft 365 MSO (Version 2206)) and Stata 17 (StataCorp. 2021. Stata Statistical Software: Release 17. College Station, TX: StataCorp LLC).

Descriptive analyses examined each dependent and independent variable’s weighted and unweighted proportions overall and by individual- and country-level characteristics. Two-way frequency tables with global chi-squared measures of association across all levels of the variables were used to determine significance of frequency differences and to inform model building. Independent variables were selected based on the literature and univariate analyses. The following variables that showed significant chi-squared test of association with any dependent variable were entered into models as covariates: NTPs use, sex, age group, nativity status, annual family income, education level, social norms, living with children, living with another person who uses NTPs, residential area, and MPOWER scores. MPOWER scores were entered into the models as continuous variables. Other covariates were entered into the models as categorical variables. Final models were adjusted for sex, age group, nativity status, annual family income, living with children, living with another person who uses NTPs, residential area, NTPs use, and MPOWER scores. Unadjusted and adjusted Poisson regression models with robust error variance were fit separately for each of the four reasons, and adjusted risk ratios (ARR) and their 95% confidence intervals from these models are presented. Goodness-of-fit was determined by deviance and Pearson goodness-of-fit tests. Statistical significance was determined by p≤0.05. Sensitivity analyses were performed by including survey year to control for the possible impact of the COVID-19 pandemic and the discrepancy of years of survey in Australia compared to other countries.

## RESULTS

### Reasons to reduce or stop nicotine and tobacco consumption

**Figure 1:**
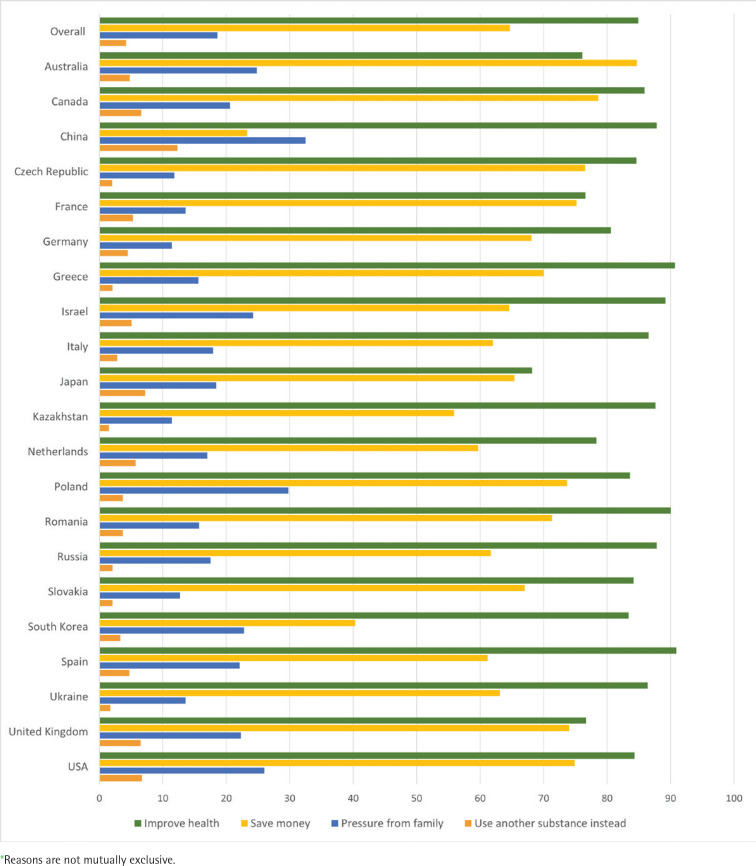
Reasons* to reduce or stop nicotine and tobacco consumption in the next 12 months among adults of legal age to use tobacco, by country, Nicotine Survey, 2019-2020 (N=82,864).

Among the options provided, the most selected reason to reduce or stop nicotine and tobacco consumption in the next 12 months was improving health (84.9%), followed by saving money (64.7%), pressure from family (18.6%), and using another substance instead (4.2%) among all survey countries aggregated. Estimates of reporting health reason were highest among respondents from Spain (90.9%), Greece (90.7%), and Romania (90%), and lowest among respondents from Japan (68.2%). In Australia, saving money was the most frequently reported reason among all countries (84.7%) and the top choice among the country residents. Estimates of saving money were the lowest in China (23.3%) and South Korea (40.3%). China had the highest estimates of selecting pressure from the family (32.5%) and using another substance instead (12.3%) compared with other countries. Pressure from the family was the second leading choice in China following saving money, which was also different from the other countries. The lowest estimates of selecting pressure from the family were observed in Kazakhstan (11.4%), Germany (11.4%), and Czech Republic (11.8%). The lowest estimates of selecting use of another substance instead were reported in Kazakhstan (1.5%) and Ukraine (1.7%) (Figure 1).

### MPOWER scores

Among the Nicotine Survey countries, from the possible range of 7–34, summary MPOWER scores ranged from 22 in Israel to 33 in Australia with a mean score of 27.3 and a median of 28 (Table 1).

**Table 1:** MPOWER scores and country characteristics of twenty-one Nicotine Survey 2019–2020 countries including WHO 2018 and World Bank 2019–2020 data (N=82864)

			*MPOWER scores*								
*Country of residence*	*Number of Nicotine Survey respondents*	*Country income level*	*Monitor*	*Protect*	*Offer*	*Warn*	*Enforce*	*Raise*	*Campaigns*	*Sum of scores*	*Average score*
Australia	2071	High-income	4	5	5	5	4	5	5	33	4.7
Canada	4033	High-income	4	5	5	5	4	4	2	29	4.1
China	4123	Middle-income	3	2	4	3	4	4	4	24	3.4
Czech Republic	4016	High-income	4	3	5	5	4	5	4	30	4.3
France	4012	High-income	4	1	4	5	4	5	5	28	4.0
Germany	4028	High-income	4	2	4	5	4	4	5	28	4.0
Greece	4093	High-income	4	5	4	5	4	5	2	29	4.1
Israel	4,059	High-income	3	3	4	3	2	5	2	22	3.1
Italy	4047	High-income	4	1	4	5	4	5	5	28	4.0
Japan	4026	High-income	4	3	4	3	2	4	3	23	3.3
Kazakhstan	4050	Middle-income	4	3	4	5	4	4	1	25	3.6
Netherlands	4011	High-income	4	2	5	5	4	4	4	28	4.0
Poland	4022	High-income	4	3	4	5	4	5	2	27	3.9
Romania	4060	Middle-income	4	5	4	5	4	4	2	28	4.0
Russia	4014	Middle-income	4	5	4	5	5	4	2	29	4.1
Slovakia	4030	High-income	4	3	5	5	4	5	2	28	4.0
South Korea	4019	High-income	4	2	5	4	2	4	5	26	3.7
Spain	4044	High-income	4	5	4	5	5	5	2	30	4.3
Ukraine	4052	Middle-income	4	4	3	5	4	4	3	27	3.9
United Kingdom	4021	High-income	4	5	4	5	4	5	5	32	4.6
USA	4033	High-income	4	2	5	4	2	3	5	25	3.6

All countries had either recent or representative data (score of 3 in China and Israel) or recent, representative, and periodic data for both adults and youth on monitoring tobacco use (score of 4).

‘Protect’ scores ranged from 1 in Italy and France, meaning that data were not reported or categorized, to 5 in Australia, Canada, Greece, Romania, Russia, Spain, and UK, meaning that all public places are completely smoke-free or at least 90% of the population are covered by complete subnational smoke-free legislation (Table 1).

‘Offer’ scores were more uniform across the survey countries ranging from 4 to 5, which means that all countries offer nicotine replacement therapy (NRT) and/or some cessation services with some cost-coverage. Only Ukraine had score of 3 for availability of NRT and/or some cessation services but no coverage of the associated costs (Table 1).

Sixteen of the survey countries reported the highest ‘Warn’ score of 5 by requiring 50% or more of a tobacco product pack to show pictures, pictograms, and health warnings. South Korea and USA require 31–49% pack coverage including pictures or pictograms (score of 4). China, Israel, and Japan require health warnings to cover ≥30% of a pack, but no pictures or pictograms (score of 3) (Table 1).

‘Campaigns’ scores ranged from 1 (data not reported) in Kazakhstan to 5 (high-quality national campaign including airing on TV and/or radio) in Australia, France, Germany, Italy, South Korea, UK, and USA (Table 1).

In Russia and Spain all forms of direct and indirect tobacco advertising are banned (‘Enforce’ score of 5). Israel, Japan, South Korea, and USA have the lowest scores of 2, meaning they have a complete absence of bans, or a ban that does not cover national television, radio, and print media. All other survey countries ban tobacco advertising on national television, radio, and print media, as well as on some but not all other forms of direct and/or indirect advertising (score of 4) (Table 1).

All countries except USA showed ‘Raise’ scores of 4 and 5 which translated into 51% or more of retail price being tax. USA taxes comprise 26–50% of retail price (score of 3) (Table 1).

### Proportion of selected reasons to reduce nicotine and tobacco product consumption by individual and country characteristics

Estimates of reported reasons to reduce or stop NTPs varied by the levels of independent variables as shown by significant chi-squared tests of association (p<0.05), with a few exceptions (Table 2). Selecting improving health as a reason to reduce or stop NTPs consumption did not differ between women and men. The proportion of selecting using another substance as a reason did not differ between residents living in rural and urban areas or between middle- and high-income countries (Supplementary file Table 1).

**Table 2:** Association of reasons to reduce or stop nicotine and tobacco product consumption with sociodemographic characteristics, nicotine and tobacco products use, and MPOWER scores among adults in twenty-one countries, Nicotine Survey 2019–2020, WHO 2018, and World Bank 2019–2020 (N=82864)

*Independent variables*	*Improve health ARR (95% CI)*	*Save money ARR (95% CI)*	*Pressure from family ARR (95% CI)*	*Use another substance instead ARR (95% CI)*
**Sex** (Ref. Women)				
Men	1.00 (0.99–1.01)	1.01 (0.99–1.03)	1.05 (0.98–1.11)	**1.32 (1.15–1.51)**
**Age** (years, Ref. 18–24)				
25–34	**1.04 (1.02–1.07)**	1.00 (0.97–1.04)	**0.87 (0.79–0.97)**	**0.81 (0.66–0.99)**
35–44	**1.07 (1.05–1.10)**	0.99 (0.95–1.02)	**0.84 (0.75–0.93)**	**0.69 (0.55–0.85)**
45–54	**1.08 (1.05–1.10)**	**0.96 (0.93–0.99)**	0.91 (0.82–1.01)	**0.59 (0.47–0.74)**
55–64	**1.08 (1.05–1.10)**	**0.94 (0.91–0.98)**	**1.15 (1.03–1.29)**	**0.65 (0.51–0.84)**
65–74	**1.06 (1.02–1.10)**	0.95 (0.88–1.01)	**1.41 (1.19–1.68)**	**0.54 (0.32–0.92)**
**Nativity status** (Ref. Native born)				
Lived in the country for <5 years	**0.68 (0.62–0.73)**	**0.78 (0.71–0.85)**	**1.70 (1.46–1.97)**	**2.17 (1.62–2.90)**
Lived in the country for ≥5 years	**0.89 (0.86–0.91)**	1.00 (0.97–1.04)	**1.25 (1.13–1.39)**	**1.63 (1.33–1.99)**
Undisclosed	**0.84 (0.77–0.91)**	**0.85 (0.75–0.96)**	1.10 (0.83–1.46)	1.13 (0.63–2.04)
**Annual family income** (Ref. Income below the national average)				
Income at or above the national average	1.01 (1.00–1.02)	**0.86 (0.84–0.87)**	**1.36 (1.28–1.45)**	**1.20 (1.04–1.39)**
**Living with children in the household** (Ref. No)				
Yes	1.00 (0.99–1.01)	0.98 (0.96–1.00)	**1.62 (1.52–1.73)**	**1.41 (1.22–1.63)**
**Living with a household member who uses nicotine or tobacco products** (Ref. no)				
Yes	1.01 (1.00–1.02)	**1.08 (1.06–1.10)**	**0.76 (0.72–0.81)**	**1.22 (1.07–1.40)**
**Residential area** (Ref: Rural area)				
Urban area	**1.04 (1.01–1.06)**	**0.95 (0.92–0.99)**	1.06 (0.94–1.19)	0.94 (0.73–1.21)
**Nicotine and tobacco product use** (Ref. Single use of cigarettes)				
Multiple NTP use	1.01 (1.00–1.02)	1.01 (0.99–1.04)	**1.26 (1.18–1.35)**	**2.81 (2.37–3.32)**
Cigarillos only	**0.86 (0.79–0.93)**	**0.78 (0.69–0.89)**	1.08 (0.83–1.41)	1.72 (0.94–3.13)
Cigars only	**0.82 (0.75–0.89)**	**0.71 (0.61–0.82)**	1.27 (0.97–1.67)	**2.32 (1.37–3.92)**
E-cigarettes only	**0.91 (0.88–0.94)**	**0.80 (0.75–0.85)**	1.01 (0.87–1.17)	**2.02 (1.39–2.93)**
Heated tobacco products only	**0.93 (0.89–0.98)**	**0.89 (0.82–0.97)**	0.80 (0.64–1.00)	0.78 (0.39–1.54)
Roll-your-own tobacco only	**0.96 (0.94–0.99)**	**0.88 (0.84–0.92)**	0.89 (0.77–1.03)	1.35 (0.94–1.93)
Shisha only	**0.88 (0.82–0.95)**	**0.64 (0.55–0.74)**	0.94 (0.72–1.22)	0.87 (0.42–1.84)
Smokeless tobacco only	**0.54 (0.46–0.64)**	**0.79 (0.68–0.92)**	**1.85 (1.46–2.34)**	**2.58 (1.52–4.38)**
**Monitor tobacco use** (continuous)	**0.80 (0.76–0.85)**	**1.17 (1.06–1.29)**	**0.80 (0.66–0.98)**	0.99 (0.59–1.67)
**Protect from tobacco smoke** (continuous)	**1.01 (1.00–1.02)**	**1.05 (1.04–1.06)**	**1.06 (1.03–1.09)**	1.07 (1.00–1.14)
**Offer help to quit tobacco** (continuous)	1.01 (1.00–1.02)	1.00 (0.98–1.02)	**1.11 (1.04–1.17)**	**1.26 (1.10–1.45)**
**Warn about the dangers of tobacco** (continuous)	**1.10 (1.06–1.13)**	**1.25 (1.19–1.32)**	**0.85 (0.76–0.95)**	**0.55 (0.42–0.74)**
**Enforce bans on tobacco advertising** (continuous)	**0.98 (0.97–0.99)**	**0.84 (0.82–0.87)**	0.97 (0.91–1.03)	**1.28 (1.11–1.47)**
**Raise taxes on tobacco** (continuous)	**0.98 (0.97–1.00)**	**1.11 (1.09–1.13)**	**1.08 (1.02–1.14)**	0.90 (0.79–1.02)
**Anti-tobacco mass media campaigns** (continuous)	**0.99 (0.98–0.99)**	1.01 (1.00–1.02)	**1.03 (1.00–1.06)**	**1.15 (1.08–1.23)**

ARR: adjusted risk ratio. Models: adjusted Poisson regression with robust error variance. Models were adjusted for sex, age group, nativity status, annual family income, living with children, living with another person who uses NTPs, residential area, NTPs use, and MPOWER scores. Bold font indicates p≤0.05. Due to rounding, some estimates may include 1.00 and have p≤0.05.

### Associations between reasons to reduce or stop NTP use, MPOWER scores, and other covariates

Associations between reasons to reduce or stop NTPs consumption and MPOWER scores, as well as associations between the reasons and other independent variables, varied in direction and strength. Even though statistically significant, many associations were very close to 1.0 (Table 2).

*Improve health*

A point-increase in a country’s ‘Monitor’ score was associated with decreased probability of reporting the health reason (adjusted risk ratio, ARR=0.80; 95% CI: 0.76–0.85). Those who have lived in a country for <5 or ≥5 years were less likely to cite the reason of improving health compared to native born residents. Those reporting multiple NTPs use or cigarette-only use also were more likely to report the health reason compared to exclusive use of any other NTPs (Table 2).

*Save money*

Higher scores on ‘Monitor’ (ARR=1.17; 95% CI: 1.06–1.29), ‘Warn’ (ARR=1.25; 95% CI: 1.19–1.32), and ‘Raise’ (ARR=1.11; 95% CI: 1.09–1.13) were associated with higher likelihood of reporting saving money as the motivation to reduce or stop NTPs consumption (Table 2). Each point increase in ‘Enforce’ score was associated with 16% lower ARR of reporting this reason (ARR=0.84, 95% CI: 0.82–0.87). Native born and those living in a country for ≥5 years were more likely to be motivated by saving money than those who lived in a country for <5 years and those with undisclosed nativity status. Lower income, smoking cigarettes exclusively, and using multiple NTPs were associated with higher ARR for citing money as a reason to reduce NTPs consumption (Table 2).

*Pressure from the family*

A higher probability of choosing pressure from the family as a reason to reduce or stop tobacco consumption was associated with increased country score for ‘Offer’ (ARR=1.11; 95% CI: 1.04–1.17) policies (Table 2). Choosing this reason was inversely associated with increased ‘Monitor’ (ARR=0.80; 95% CI: 0.66–0.98) and ‘Warn’ (ARR=0.85; 95% CI: 0.76–0.95) scores. Compared to participants aged 18–24 years, the age groups 25–34 and 35–44 years were less likely, and ≥55 years age group more likely to cite family pressure as motivation to reduce NTPs consumption. Immigrant status, higher income, living with children, and not living with those who used NTPs were also associated with a higher probability of choosing family pressure as the reason. Compared to exclusive cigarette use, use of multiple NTPs and exclusive use of smokeless tobacco had higher probability for citing family pressure (Table 2).

*Use another substance instead*

Higher probability of selecting use of another substance as a reason to reduce or stop NTP use was associated with increased country scores of ‘Offer’ (ARR=1.26; 95% CI: 1.10–1.45), ‘Enforce’ (ARR=1.28; 95% CI: 1.11–1.47), and ‘Campaigns’ (ARR=1.15; 95% CI: 1.08–1.23) policies (Table 2). A higher country score of ‘Warn’ was associated with decreased probability of choosing this reason (ARR=0.55; 95% CI: 0.42–0.74). Men compared to women, people who have lived in a country for <5 or ≥5 years compared to native born residents; those with income above the national average compared to those with income below the national average; those who lived with children aged <18 years, and those who lived with other household members who used NTPs compared to the respondents who were not, were more likely to be motivated by using another substance instead. The youngest age group was more likely to be motivated by using another substance instead of tobacco compared to any other age group. Compared to respondents who only smoked cigarettes, respondents who used multiple NTPs, or only used cigars, e-cigarettes or SLT, were also more likely to cite this reason (Table 2).

Sensitivity analyses additionally controlled for survey year showed results similar to the original models. For example, a point increase in ‘Warn’ score was associated with ARR of 1.25 (95% CI: 1.19–1.32) for saving money, 0.85 (95% CI: 0.76–0.95) for family pressure, and 0.55 (95% CI: 0.42–0.74) for using another substance instead (data not shown in tables).

## DISCUSSION

The findings of this study suggest that improving health, saving money, and responding to pressure from the family are the most prevalent reasons for adults who plan to reduce or stop nicotine and tobacco consumption in the next 12 months. The proportion of respondents reporting improving health, saving money, and family pressure as reasons to reduce or quit tobacco varied by sociodemographic factors, type of NTPs used, and MPOWER scores in the respondents’ country of residence.

The top three reasons cited by Nicotine Survey respondents concur with findings from other studies that find health, money, and family concerns to be leading motivations to quit tobacco consumption^7,11,13^. Other reasons such as social pressure, improving personal fitness, illness of a friend or family member, and negative self-image are also mentioned in the literature and deserve examination by future studies^8,11,13^. The two most frequent reasons reported to reduce or quit NTPs in the present study were to improve health and save money. Only two countries were different from the rest: Australia had a higher proportion of selecting saving money than improving health, and China had a higher reported proportion of pressure from family than saving money. These disparities are notable, indicating potential cultural^18^ and economic^19,20^ differences essential to the motivation behind quitting or reducing NTPs consumption. For example, Australia’s policies of higher taxation make its cigarette prices the highest in the world, which may have contributed to saving money being the more prevalent reason to quit cigarette consumption^21^. Survey respondents had an opportunity to select ‘none of the above’ or ‘other’ reasons to reduce or quit NTPs consumption, however <4% selected either of these reasons. This suggests that people who intend to quit tobacco consumption are mostly motivated by health, costs, and family pressure. Intention to use another substance instead was selected only by 4.2% of Nicotine Survey respondents overall, although it reached 12.3% among China respondents.

The Nicotine Survey did not specify what substance was meant to be used to replace nicotine or tobacco, leaving this interpretation to the survey respondents. Transitions may include switching from combustible to non-combustible tobacco^22,23^, heated tobacco products^24,25^, nicotine replacement products^26-28^, or cannabis^29^. However, current research on transitions from NTPs is mostly focused on adolescents and a limited range of substances. Future research might discover additional NTPs substitutes among adults.

Understanding what motivates people to reduce or quit tobacco and how this motivation may be associated with MPOWER scores can help focus a country’s tobacco control policies. Existing studies show that tobacco control policies are associated with increased intention to quit and quit attempts but did not specifically examine the reasons to do so^2,6^. This study elucidates that intention to quit because of concerns for health, money, and family was also associated with level of implementation of the MPOWER policies. MPOWER scores use a standardized approach and are routinely collected by WHO in all countries regardless of their ratification of its Framework Convention for Tobacco Control (FCTC). In the case of this study, USA is a Nicotine Survey country that signed but did not ratify WHO FCTC and is not accountable to follow MPOWER. However, having WHO data for USA’s MPOWER scores allowed us to keep it in the study.

Only one MPOWER policy score – ‘Protect’ – was consistently positively associated with three out of four reported reasons to reduce or quit NTPs consumption (health, money, and family). However, the strength of this association was not sufficient to make any practical inferences. Four MPOWER scores (Monitor, Protect, Warn, and Raise) were significantly positively associated with saving money as the reason to reduce or quit NTPs consumption. The strongest association of ‘Raise’ policy with reported reasons was with motivation by saving money, which is supported by other peer-reviewed research^7,12^. This association was even more evident in the case of Australia where prices of cigarettes are the highest globally^30^.Each MPOWER score was significantly positively associated with at least one reason to reduce or quit NTPs consumption. While ‘Offer’ was not significantly associated with either improving health or saving money, it was significantly positively associated with pressure from family and use of another substance as reasons to quit or reduce NTPs consumption. This association can be particularly important for countries like China, where pressure from family was reported as a more common reason to reduce or quit. ‘Enforce’ score was also not positively associated with improving health or saving money; however, it was significantly positively associated with use of other substances as a reason to quit or reduce NTPs consumption. ‘Campaigns’ score had an inverse association with health but positive associations with family pressure and using another substance, which may be related to country-specific contents of the campaigns, their reach, and target audience^13^. Further research should consider examining both positive and negative associations of MPOWER scores to understand the relationship to pragmatic reasons people are reducing or quitting.

### Limitations

The study is subject to some limitations. First, this ecologic study using secondary data examines associations among variables from different datasets. Second, the online opt-in panel sample of the survey is not nationally representative of the overall tobacco users in each country. Third, many questions required respondents to recall their behavior in the recent month, which might have resulted in recall bias. Fourth, response options for reasons to reduce or stop nicotine and tobacco consumption in the next 12 months were pre-determined and may not have captured other factors contributing to the reasoning. For example, the response option ‘use another substance instead’ in the Nicotine Survey was open to interpretation by the respondent and could potentially include a range of substances from different forms of nicotine and tobacco to nicotine replacement products to illicit drugs, leading to possible overestimation of the reported proportion for selecting this reason. Fifth, MPOWER measures have been developed around cigarettes and only partially include non-cigarette tobacco products in some countries. In this study’s sample (data not presented in tables), 69% of respondents who used any NTPs had used cigarettes at least monthly exclusively or in combination with other NTPs, and 31% used only non-cigarette NTPs. It is difficult to assess how MPOWER measures that are based on cigarette consumption may reach and affect those who consume non-cigarette tobacco products. Finally, analysis of confounding factors was limited due to the availability of comparable country-level data. Further research should examine potential confounding factors that have resulted in the inverse associations between MPOWER scores and the reasons to reduce or quit NTPs consumption. For example, Monitor was significantly associated with improve health, save money, and pressure from the family reasons. However, it is unlikely that conducting regular national-level surveys on tobacco use impacts an individual’s motivation to quit NTPs. It is, therefore, likely these associations result from a confounding factor and yield an opportunity for further research.

## CONCLUSIONS

Improving health, saving money, and pressure from the family were the most prevalent reasons to reduce or quit NTP consumption in the next 12 months among adults using such products at least monthly. Some MPOWER scores showed association with increased motivation to reduce or stop nicotine and tobacco consumption to improve health, save money, respond to family pressure, or use another substance instead. Strengthening these policies may help reduce the demand for tobacco products by enhancing people’s motivation to reduce or quit tobacco consumption.

## Data Availability

The data supporting this research are available from the following source: https://www.euromonitor.com/usa
