# Supplementary material for: Reasons to decrease or stop nicotine and tobacco use among adults and association with MPOWER scores in twenty-one middle- and high-income countries, 2019–2020
Source: Tob Prev Cessat. 2023 Jul 21;9:25. doi: 10.18332/tpc/167957 (PMC10360442; doi:10.18332/tpc/167957)
Supplement: Supplementary file 1 [file TPC-9-25-s1.pdf]

Supplementary attachment 1

Definitions of nicotine and tobacco products in Nicotine Survey 2019-2020

Cigarettes: A roll of cut tobacco machine-manufactured into a paper cylinder for the purpose of smoking. Common examples include Marlboro, Newport, Camel, Pall Mall, and L&M.

Hand-rolled/RYO (roll your own) /MYO (make your own) tobacco: Cut tobacco sold in packaged format for smoking either in roll-your-own (RYO) cigarettes (rolled by hand using loose cigarette paper) or make-your-own (MYO) cigarettes (inserted in filter tubes manually or using a machine). Includes volume tobacco. Common examples include Bugler, Natural American Spirit, Drum, and Zig Zag.

Smokeless tobacco: Smokeless tobacco is used either in the mouth or in the nose, by chewing, sucking or inhaling (in the case of nasal snuff) and traditionally has been divided into two subcategories, snuff and chewing tobacco. Snuff was originally a nasal product but today is more commonly used in the mouth (oral snuff, moist snuff) in a manner similar to that of chewing tobacco. Common examples include Copenhagen, Grizzly, and Skoal.

Cigars: Cigars are made from tobacco wrapped in leaf as opposed to paper (such as cigarettes). Cigars generally consist of three sections: the filler, the binder and the wrapper though the product varies considerably in terms of price, quality and size. Different terms are used to describe the various types of cigar depending on country. So-called ‘blunt wraps’, made from homogenised tobacco, are excluded. Common examples include Dutch Masters, Garcia y Vega, and Montecristo.

Cigarillos: Cigarillos are defined as miniature cigars weighing less than 3 grams each, with a ring gauge of ≤29. Can be unfiltered and unflavoured but more commonly contain filter, aroma or both. Common examples include Swisher Sweets, Black & Mild, and White Owl.

Shisha/Hookah/Pipe tobacco: Includes water pipe tobacco originating in the Middle East, known as 'shisha', hookah and narguile. Typically consumed away from home in public, social spaces. Also includes medwakh/dokha tobacco. Common examples include Al Fakher, Nakhla, and Starbuzz.

E-cigarettes (does not include traditional cigarettes): An electronic device used to vapourise liquid nicotine. Includes products with a cartridge, pod system, or refillable tank system. Types of e-cigarettes may be: cig-a-likes (an e-cigarette product which are intended to mimic traditional rolled manufactured cigarettes and which contain a battery and enclosed flavour cartridge), non-cig-a-like closed systems (an e-cigarette product which do not physically resemble cigarettes but which incorporate the use of enclosed flavour refill pods or cartridges e.g. Juul), and products with a refillable e-liquid chamber and comprise a power source (battery), a tank to hold e-liquid (vapouriser) and e-liquid itself. Common examples include JUUL, Vuse, Blu, SMOK, Innokin, and Eleaf.

Heated tobacco (does not include cigarettes, cigars, cigarillos, etc.): Products, generally manufactured by major tobacco companies, which heat rather than combust tobacco to produce vapour rather than smoke e.g. iQOS, glo, Lil/Fiit.

Supplementary attachment 2

Definitions of MPOWER scores from WHO MPOWER Overview Data by country (2018)

(available at https://apps.who.int/gho/data/node.main.TOBMPOWER?lang=en)

Monitor (1-4)

1 = No known data or no recent* data or data that are not both recent* and representative** 2 = Recent* and representative** data for either adults or youth 3 = Recent* and representative** data for both adults and youth 4 = Recent*, representative** and periodic*** data for both adults and youth

* Recent means in the data year or the 5 years previous to the data year. ** Survey sample representative of the national population. *** Survey repeated at least every five years. Survey is considered an "adult survey" if it is a household survey sampled from the general population with respondent ages not limited to those under 15.

Protect from tobacco smoke (1-5)

1 = Data not reported/not categorized* 2 = Up to two public places completely smoke-free 3 = Three to five public places completely smoke-free 4 = Six to seven public places completely smoke-free 5 = All public places completely smoke-free (or at least 90% of the population covered by complete subnational smoke-free legislation)

Country's legislation is assessed to determine whether smoke-free laws exist in each of the following places at either the national or subnational level: • health-care facilities; • educational facilities other than universities; • universities; • government facilities; • indoor offices; • restaurants; • pubs and bars; • public transport. *In several countries, in order to significantly expand the creation of smoke-free places, including restaurants and bars, it was politically necessary to include exceptions to the law that allowed for the provision of designated smoking rooms. The requirements for designated smoking rooms are so technically complex and stringent that, for practical purposes, few or no establishments are expected to implement them. Because no data were requested on the number of complex designated smoking rooms actually constructed, it is not possible to know whether these laws have resulted in the complete absence of such rooms, as intended. For this reason, these few countries have not been categorized in the analyses for this indicator.

Offer help to quit tobacco use (1-5)

1 = Data not reported 2 = None 3 = NRT* and/or some cessation services** (neither cost-covered) 4 = NRT* and/or some cessation services** (at least one of which is cost-covered) 5 = National quit line, and both NRT* and some cessation services** cost-covered

* Nicotine replacement therapy. ** Smoking cessation support available in any of the following places: health clinics or other primary care facilities, hospitals, office of a health professional, the community.

Warn about the dangers of tobacco (1-5)

1 = Data not reported 2 = No warning or warning covering <30% of pack surface 3 = ≥30%* but no pictures or pictograms and/or other appropriate characteristics** 4 = 31%–49%* including pictures or pictograms and other appropriate characteristics** 5 = ≥50%* including pictures or pictograms and appropriate characteristics**

* average of the front and back of the cigarette pack. ** • Specific health warnings mandated; • appearing on individual packages as well as on any outside packaging and labelling used in retail sale; • describing specific harmful effects of tobacco use on health; • are large, clear, visible and legible (e.g. specific colours and font style and sizes are mandated); • rotate; • written in (all) principal language(s) of the country

Enforce bans on tobacco advertising (1-5)

1 = Data not reported 2 = Complete absence of ban, or ban that does not cover national television (TV), radio and print media 3 = Ban on national TV, radio and print media only 4 = Ban on national TV, radio and print media as well as on some but not all other forms of direct* and/or indirect** advertising 5 = Ban on all forms of direct* and indirect** advertising.

* Direct advertising bans: • national television and radio; • local magazines and newspapers; • billboards and outdoor advertising; • point of sale. ** Indirect advertising bans: • free distribution of tobacco products in the mail or through other means; • promotional discounts; • non-tobacco products identified with tobacco brand names (brand extension); • brand names of non-tobacco products used for tobacco products; • appearance of tobacco products in television and/or films; • sponsored events.

Raise taxes on tobacco (1-5)

1 = Data not reported 2 = ≤ 25% of retail price is tax 3 = 26–50% of retail price is tax 4 = 51–75% of retail price is tax 5 = >75% of retail price is tax.

Taxes assessed include excise tax, value added tax (“VAT”), import duty (when the cigarettes were imported) and any other taxes levied. Only the price of the most popular brand of cigarettes is considered. In the case of countries where different levels of taxes applied to cigarettes are based on either length, quantity produced or type (e.g. filter vs. non-filter), only the rate that applied to the most popular brand is used in the calculation. Given the lack of information on country and brand-specific profit margins of retailers and wholesalers, their profits were assumed to be zero (unless provided by the national data collector).

Anti-tobacco mass media campaigns (1-5)

1 = Data not reported 2 = No national campaign conducted in the reporting period with a duration of at least three weeks 3 = National campaign conducted with 1-4 appropriate characteristics* 4 = National campaign conducted with 5-6 appropriate characteristics*, or with 7 characteristics excluding airing on TV and/or radio 5 = National campaign conducted with at least 7 appropriate characteristics* including airing on TV and/or radio.

* Characteristics of a high-quality campaign are: • the campaign was part of a tobacco control programme; • before the campaign, research was undertaken or reviewed to gain a thorough understanding of the target audience; • campaign communications materials were pretested with the target audience and refined in line with campaign objectives; • air time (radio, television) and/or placement (billboards, print advertising, etc.) was obtained by purchasing or securing it using either the organization’s own internal resources or an external media planner or agency (this information indicates whether the campaign adopted a thorough media planning and buying process to effectively and efficiently reach its target audience); • the implementing agency worked with journalists to gain publicity or news coverage for the campaign; • process evaluation was undertaken to assess how effectively the campaign had been implemented; and • an outcome evaluation process was implemented to assess the campaign impact.

Supplementary table 1

Proportion of selected reasons* to reduce nicotine and tobacco product consumption among adults in 21 countries, by individual and country characteristics, Nicotine Survey 2019-2020, WHO 2018, and World Bank 2019-2020

	**Improve health**		**Save money**		**Pressure from family**		**Use another substance instead**	
	**Unwtd freq**	**Wtd% (95% CI)**	**Unwtd freq**	**Wtd% (95% CI)**	**Unwtd freq**	**Wtd% (95% CI)**	**Unwtd freq**	**Wtd% (95% CI)**
**Overall**	18,595	86.5 (85.8-87.3)	14,194	64.7 (64.0-65.4)	4,081	18.6 (18.1-19.2)	939	4.2 (3.9-4.5)
**Nicotine and tobacco products (NTPs) use**								
Multiple NTPs use	**7,370**	**86.5 (85.8-87.3)**	**5,737**	**67.1 (66.1-68.2)**	**1,822**	**21.4 (20.5-22.4)**	**592**	**6.8 (6.2-7.3)**
A single NTP use only (any NTP)	**11,226**	**83.9 (83.2-84.5)**	**8,458**	**63.2 (62.3-64.1)**	**2,260**	**16.9 (16.2-17.5)**	**347**	**2.6 (2.3-2.8)**
Cigarettes only	**8,387**	**86.9 (86.2-87.6)**	**6,357**	**66.0 (64.9-67.0)**	**1,592**	**16.7 (15.9-17.5)**	**203**	**2.0 (1.7-2.3)**
Cigarillos only	**171**	**70.8 (65-76.7)**	**127**	**52.3 (45.7-59.0)**	**51**	**20.5 (15.1-25.9)**	**11**	--
Cigars only	**159**	**66.9 (60.8-73.0)**	**110**	**46.3 (39.7-52.9)**	**52**	**21.3 (15.6-27.0)**	**15**	**6.3 (3.2-9.5)**
E-cigarettes only	**767**	**76.7 (73.9-79.6)**	**542**	**53.3 (49.8-56.7)**	**189**	**18.1 (15.6-20.5)**	**45**	**5.1 (3.4-6.9)**
Heated tobacco products only	**361**	**77.8 (73.9-81.7)**	**274**	**59.4 (54.7-64.1)**	**72**	**14.6 (11.4-17.8)**	**9**	--
Roll-your-own tobacco only	**1,097**	**82.1 (79.8-84.3)**	**842**	**62.4 (59.6-65.3)**	**196**	**14.2 (12.3-16.1)**	**39**	**2.7 (1.9-3.6)**
Shisha only	**208**	**72.3 (66.6-77.9)**	**117**	**40.4 (34.4-46.4)**	**48**	**16.5 (12.1-21.0)**	**9**	--
Smokeless tobacco only	**76**	**41.7 (34.1-49.2)**	**89**	**51.8 (44.1-59.6)**	**60**	**35.2 (27.7-42.7)**	**16**	**9.2 (4.8-13.7)**
**Sex**								
Women	8419	85.3 (84.6-86.1)	**6,528**	**65.7 (64.7-66.8)**	**1,696**	**17.2 (16.4-18.0)**	**357**	**3.4 (3.0-3.8)**
Men	10,151	84.7 (84.1-85.4)	**7,641**	**63.9 (63.0-64.8)**	**2,368**	**19.7 (19-20.5)**	**575**	**4.8 (4.3-5.2)**
**Age group (years)**								

18-24	**2,222**	**78.0 (76.4-79.6)**	**1,893**	**65.5 (63.7-67.4)**	**521**	**18.1 (16.6-19.6)**	**172**	**6.3 (5.3-7.2)**
25-34	**4,111**	**83.3 (82.2-84.3)**	**3,226**	**65.9 (64.5-67.2)**	**893**	**18.3 (17.2-19.4)**	**270**	**5.4 (4.8-6.1)**
35-44	**4,448**	**86.4 (85.4-87.4)**	**3,349**	**65.5 (64.1-66.8)**	**939**	**18.3 (17.3-19.4)**	**212**	**4.2 (3.6-4.7)**
45-54	**4,093**	**87.0 (86.0-87.9)**	**3,002**	**63.8 (62.3-65.2)**	**850**	**18.1 (17.0-19.2)**	**149**	**3.2 (2.7-3.7)**
55-64	**2,983**	**87.3 (86.1-88.4)**	**2,180**	**63.5 (61.8-65.2)**	**670**	**19.1 (17.7-20.5)**	**106**	**3.0 (2.4-3.5)**
65-74	**738**	**85.5 (82.8-88.2)**	**544**	**62.5 (58.5-66.5)**	**208**	**21.8 (18.4-25.2)**	**30**	**2.4 (1.3-3.5)**
**Nativity status**								
Lived in the country for less than 5 years	**257**	**54.6 (49.9-59.2)**	**237**	**50.0 (45.3-54.6)**	**142**	**29.6 (25.3-33.8)**	**48**	**10.4 (7.5-13.2)**
Lived in the country for more than 5 years	**1,216**	**75.5 (73.4-77.7)**	**1,056**	**65.4 (62.9-67.8)**	**372**	**23.0 (20.9-25.2)**	**110**	**6.9 (5.6-8.2)**
Native born	**16,960**	**86.6 (86.1-87.1)**	**12,767**	**65.1 (64.4-65.8)**	**3,518**	**18.0 (17.4-18.6)**	**768**	**3.8 (3.5-4.1)**
Undisclosed	**162**	**67.6 (61.5-73.7)**	**134**	**54.0 (47.4-60.7)**	**49**	**20.0 (14.8-25.2)**	**13**	**4.7 (2.1-7.4)**
**Annual family income**								
Income below the national average	**9,774**	**84.2 (83.5-84.9)**	**8,200**	**70.3 (69.4-71.2)**	**1,783**	**15.4 (14.7-16.1)**	**411**	**3.4 (3.1-3.8)**
Income at or above the national average	**8,821**	**85.7 (85-86.4)**	**5,994**	**58.4 (57.4-59.4)**	**2,298**	**22.3 (21.4-23.1)**	**528**	**5.0 (4.6-5.4)**
**Education**								
Primary school graduate	**400**	**80.9 (77.4-84.4)**	**355**	**71.1 (66.9-75.3)**	**75**	**15.4 (12.1-18.8)**	**21**	**4.1 (2.4-5.9)**
Secondary school graduate	**3,131**	**83.7 (82.5-85.0)**	**2,691**	**71.5 (70.0-73.1)**	**535**	**14.5 (13.2-15.7)**	**138**	**3.8 (3.1-4.5)**
A-level or college equivalent	**408**	**76.3 (72.6-80.0)**	**409**	**77.1 (73.5-80.7)**	**84**	**15.5 (12.4-18.6)**	**35**	**6.8 (4.6-9.1)**
Vocational school graduate or technical school certificate	**5,629**	**84.8 (83.8-85.7)**	**4,499**	**67.2 (66.0-68.4)**	**1,140**	**17.2 (16.2-18.2)**	**232**	**3.5 (3.0-3.9)**
Associate's or "2-year" degree from junior college or technical school	**143**	**84.6 (79-90.2)**	**118**	**68.7 (61.4-76.1)**	**48**	**28.0 (21.2-34.9)**	**19**	**11.5 (6.6-16.4)**
Bachelor's degree from college or university	**5,940**	**86.7 (85.8-87.5)**	**3,981**	**58.4 (57.1-59.6)**	**1,480**	**21.4 (20.4-22.5)**	**345**	**4.7 (4.2-5.3)**
Graduate or post-graduate studies	**2,509**	**84.7**	**1,809**	**61.6**	**640**	**21.6**	**130**	**4.3**

(e.g., master's, doctoral degree)		**(83.2-86.1)**		**(59.7-63.5)**		**(20-23.2)**		**(3.5-5.0)**
Other	**435**	**85.6 (82.5-88.8)**	**332**	**64.8 (60.4-69.1)**	**79**	**15.3 (12-18.5)**	**19**	**3.2 (1.7-4.7)**
**Residential area**								
Rural	**1,391**	**80.6 (78.6-82.5)**	**1,220**	**70.2 (67.9-72.5)**	**282**	**16.7 (14.7-18.6)**	68	3.7 (2.8-4.6)
Urban	**17,204**	**85.3 (84.8-85.8)**	**12,974**	**64.2 (63.5-64.9)**	**3,799**	**18.8 (18.2-19.4)**	871	4.2 (3.9-4.5)
**Social norms**								
Tobacco use is not normal	**2,046**	**85.8 (84.3-87.3)**	**1,491**	**61.7 (59.6-63.9)**	**619**	**26.8 (24.7-28.8)**	**115**	**4.6 (3.7-5.4)**
Tobacco use is somewhat normal	**15,367**	**84.5 (84-85.1)**	**11,679**	**64.2 (63.5-65)**	**3,227**	**17.6 (17-18.2)**	**731**	**3.9 (3.7-4.2)**
Tobacco use is normal	**1,182**	**88.4 (86.7-90.1)**	**1,024**	**76.4 (74.1-78.8)**	**235**	**17.1 (15-19.2)**	**93**	**6.6 (5.2-7.9)**
**Living with children in the household**								
No	**10,719**	**84.4 (83.7-85.0)**	**8,368**	**65.4 (64.5-66.3)**	**1,957**	**15.5 (14.8-16.2)**	**451**	**3.4 (3.1-3.8)**
Yes	**7,876**	**85.7 (85.0-86.5)**	**5,826**	**63.6 (62.6-64.7)**	**2,124**	**23.2 (22.3-24.1)**	**488**	**5.2 (4.8-5.7)**
**Living with a household member who uses nicotine or tobacco products**								
No	9,941	84.3 (83.6-85)	**7,342**	**62.3 (61.4-63.3)**	**2,466**	**20.9 (20.1-21.7)**	**463**	**3.8 (3.5-4.2)**
Yes	8,163	85.4 (84.6-86.1)	**6,581**	**68.5 (67.6-69.5)**	**1,532**	**15.9 (15.2-16.7)**	**453**	**4.7 (4.2-5.1)**
**Country-level characteristics**								
**Country income level**								
High-income	**12,643**	**83.6 (82.9-84.2)**	**10,364**	**68.4 (67.6-69.2)**	**2,912**	**19.1 (18.5-19.8)**	662	4.3 (4.0-4.7)
Middle-income	**5,952**	**87.9 (87.0-88.7)**	**3,830**	**56.5 (55.3-57.8)**	**1,169**	**17.4 (16.4-18.5)**	277	3.9 (3.4-4.3)

*Selected reasons are not mutually exclusive
UnWtd freq – unweighted frequency
Wtd% (95% CI) – weighted percent and 95% confidence interval
Bold font – **global** chi squared tests of association across all levels of each variable are significant ($p \leq 0.05$)
-- – estimates with relative standard error of 30% or more were suppressed

© 2023 Sharapova S. R. et al.
